# Editorial: New perspectives on DCB for treatment of acute and chronic coronary artery disease

**DOI:** 10.3389/fcvm.2026.1840114

**Published:** 2026-04-14

**Authors:** Tuomas T. Rissanen, Matthias Bossard

**Affiliations:** 1 Heart Center, North Karelia Central Hospital, Siunsote, Joensuu, Finland; 2 Faculty of Health Sciences, University of Eastern Finland, Kuopio, Finland; 3 Cardiology Division—Heart Center, Luzerner Kantonsspital, Lucerne, Switzerland; 4 Faculty of Health Sciences and Medicine, University of Lucerne, Lucerne, Switzerland

**Keywords:** calcified nodules, calcium modification, *de novo*, dissection, drug-eluting balloon

Despite technological and procedural advancements, such as the use of intravascular imaging, the implantation of drug-eluting stent (DES) is associated with several limitations such as stent underexpansion and the side vessel compromise. The stent-related adverse events accumulate without a plateau, and the risk is increased by the stent length as well as by anatomical and clinical complexity. DCBs deliver antiproliferative agents without leaving a permanent metallic implant, offering the potential to reduce stent burden, preserve native vessel physiology, allowing positive vessel remodeling and shortened antiplatelet therapy (1).

**Figure 1:**
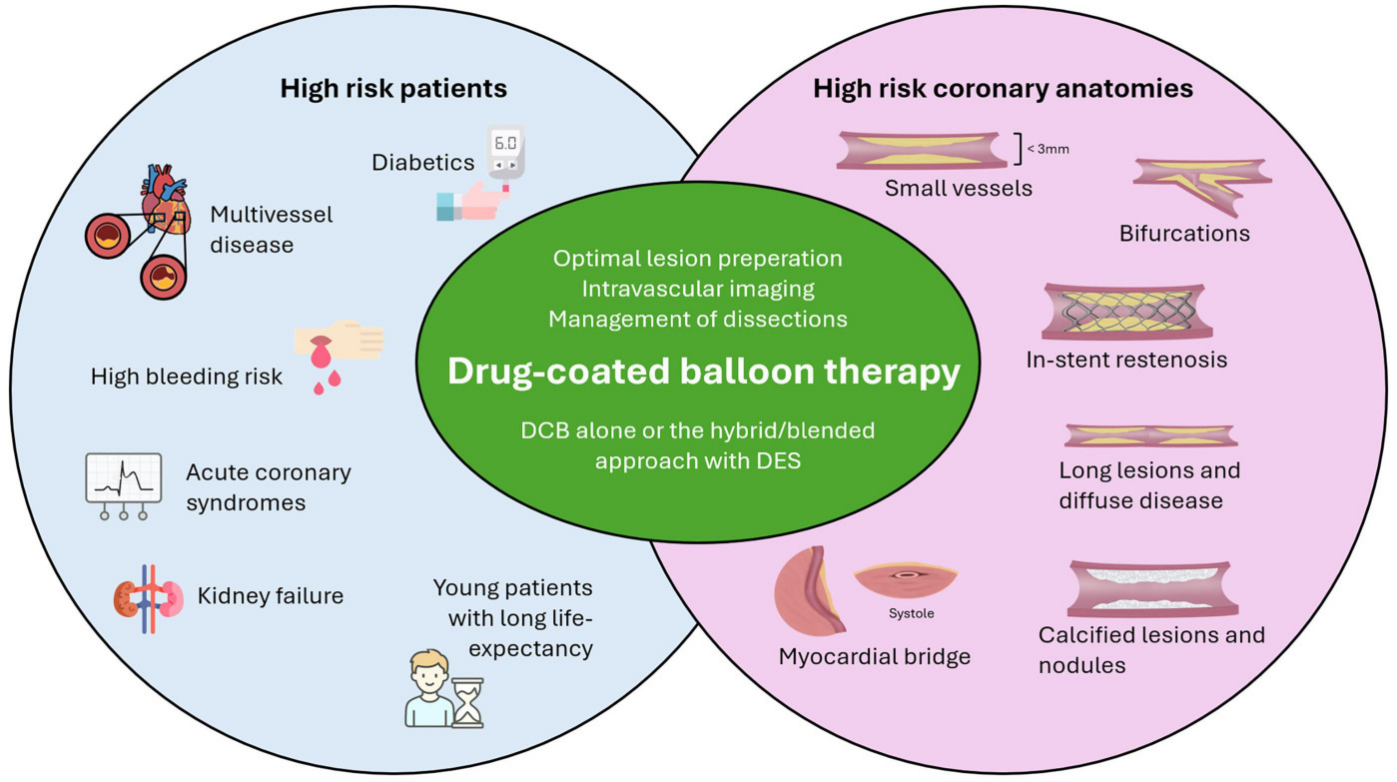
The established and potential indications for drug-coated balloon (DCB) therapy for coronary artery disease. Optimal lesion preparation, calcium modification and intravascular imaging are essential for efficacy while the management of dissections is crucial for the safety of DCB therapy.

DCBs are now an established therapy for *de novo* lesions in small vessels and in in-stent restenosis (ISR). Along with the accumulating data, operator experience and introduction of improved catheter designs, and new drug formulations, including limus-based DCBs, has led to emerging indications including treatment of large-vessels, bifurcation lesions and diffusely diseased and calcified vessels (1). Moreover, percutaneous coronary intervention (PCI) with DCB has gained interest among specific patient populations, where metallic stents have a high tendency to fail, such as in diabetics, or in patients who do not tolerate intensive antiplatelet inhibition due to high-bleeding risk (Figure 1).

In this special topic of *New Perspectives on Drug-coated Balloons (DCB) For Treatment of Acute and Chronic Coronary Artery Disease* in Frontiers in Cardiovascular Medicine, an interesting and insightful series of papers, including four research articles, a review article, a perspective and a patient case, have been published shedding light on the expanding use and new opportunities offered by DCB-PCI for management of atherosclerotic lesions.

While the treatment of small vessel disease is a well-established indication for DCB (1), the safety and utility of DCB-PCI only for treatment of large vessels and complex coronary anatomy remains debated and is considered to represent the last frontier of DCB therapy. In this context, Dr. Gitto et al. provide a comprehensive review on DCB PCI in challenging anatomical (long lesions, bifurcations, calcified vessels, thrombotic vessels) and clinical scenarios (such as high bleeding risk and diabetes) and provide practical tips and tricks for optimal *leaving nothing behind* therapy or the hybrid approach combining both DCB and DES.

As DCB-only PCI is rapidly spreading, the quality of stentless PCI is very important and comprises of both the efficacy and safety the procedure. The lesion preparation before DCB delivery focuses on maximizing lumen gain while avoiding vessel-threatening dissections. Interventional cardiologists need education on the optimal lesion preparation, and which dissections can be left behind and which must be stented. These very important aspects are discussed in this issue by. The authors provide a practical safety checklist for the operator as well as a simplified classification of dissections. Classically, coronary dissections have been classified by the National Heart, Lung and Blood Institute (NHLBI) system (A to E) which may be considered too complex for DCB-angioplasty (2). Here, a simplified two-tier dissection classification is proposed: type 1 dissections which are safe to leave, while type 2 dissections require further treatment, or even coverage with a stent or scaffold. The type 1 dissections are characterized by TIMI 3 flow and rapid contrast clearance without significant luminal compromise. The type 2 dissections include reduced flow, persistent contrast staining, spiraling dissections, or progressive lumen narrowing. By following these criteria, the authors report a very low acute vessel closure rate of 0.2% with DCB-only PCI in 10922 lesions as compared to 0.3% stent thrombosis rate, supporting the safety of this strategy when performed carefully Corballis et al.

Coronary calcium presents a significant challenge for PCI using DES because of the risk of stent underexpansion and malapposition resulting in recurrent ischemic adverse events and poor long-term outcomes. As PCI is increasingly performed on elderly patients, as well as patients with renal failure and diabetes, the prevalence of severe coronary calcification is increasing. To this end, Räsänen et al. investigated a novel approach, i.e., intravascular lithotripsy followed by paclitaxel DCB treatment, in a small cohort of 34 elderly patients with severe coronary calcification and the majority being at high bleeding risk. This new therapeutic strategy to tackle calcified lesions resulted in a low rate (3%) of clinically indicated target lesion revascularization (TLR) at one year without safety concerns. Larger observational and randomized trials are warranted on this promising approach for management of severely calcified coronary artery disease.

Calcified nodules are regarded as one of the most challenging targets in PCI. Despite major advancements in PCI, including improved devices for lesion preparation, they continue to be associated with high rates of target lesion failure (TLF). Funatsu et al. present a case of recurrent restenosis after calcium modification and DCB treatment of a calcified nodule (CN). Only after the third and most effective ablation of the CN, a durable long-term outcome was achieved. This educational case illustrates two important considerations. Firstly, the CN—particularly the eruptive subtype—should be removed as thoroughly as possible before PCI to prevent recurrent events due to its biologically active nature. Secondly, DCB treatment of a CN preserves the option for further calcium modification with all available tools should restenosis occur.

To further investigate the problem of CN, in this issue Matsuda et al. report a retrospective analysis of 68 *de novo* eruptive CNs in 58 patients who underwent DCB-only PCI optimized by optical coherence tomography (OCT) imaging. Almost all lesions (97%) required calcium-modifying devices for lesion preparation prior to DCB treatment, highlighting the complexity of eruptive CN lesions. Target lesion failure (TLF) was significantly associated with the absence of medial dissection in post-PCI OCT evaluation which corroborates with previous findings in non-calcified lesions. Also, a larger eruptive CN angle on pre-PCI OCT correlated with a higher likelihood of TLF. CN protrusion was the most common restenosis pattern followed by suboptimal lumen expansion and layered plaques.

In the myocardial bridge, a stent implantation is strongly discouraged because of an increased risk of stent fracture and failure. Wu et al. present an observational study comprising 231 patients with proximal LAD disease associated with a myocardial bridge treated with PCI using either DCB or DES and optimized using intravascular ultrasound (IVUS). DCB-angioplasty was associated with significantly lower rate of late lumen loss, TLR and MACE compared to drug-eluting stents (DES) at 12 months. Although DES produced a larger acute lumen gain, this did not translate into better clinical outcomes compared with DCB therapy. Restenosis rates were lower after DCB treatment (3.6%) than after DES implantation (13.5%). Importantly, DCB therapy allowed for shorter dual antiplatelet therapy (DAPT) duration (1 month), which was safe and not associated with increased thrombotic events. This analysis delivers a noteworthy signal that may contribute to establishing future standards for the management of lesions associated with myocardial bridges.

The restenosis rate after DES implantation in diabetic patients is high, making it the Achille's heel of PCI as compared to coronary artery bypass grafting. The adverse events are particularly high associated with diffuse disease or small vessels. The retrospective study by Sella et al. is a direct comparison between intravascular brachytherapy (IVB) and DCB for the treatment of ISR in patients with diabetes. The study demonstrated that DCB resulted in significantly lower TLF than IVB (5.2% vs. 21.3%). Moreover, target-vessel myocardial infarction rate was lower in the DCB group. Considering the better clinical outcomes, DCB or implantation of another DES should remain the standard of care of ISR also in the future.

Collectively, the contributions in this special issue shed new light on how DCB-only PCI can be applied in clinical and anatomical situations that have traditionally posed major obstacles to metallic stent implantation. We believe these reports will spark innovation and further research in the expanding arena for implant-free PCI. Finally, DCB and DES should be seen as complementary, not mutually exclusive, therapeutic tools for optimal coronary revascularization.
